# Two Decades of Research Using Taiwan’s National Health Insurance Claims Data: Bibliometric and Text Mining Analysis on PubMed

**DOI:** 10.2196/18457

**Published:** 2020-06-16

**Authors:** Sheng-Feng Sung, Cheng-Yang Hsieh, Ya-Han Hu

**Affiliations:** 1 Division of Neurology, Department of Internal Medicine Ditmanson Medical Foundation Chiayi Christian Hospital Chiayi City Taiwan; 2 Department of Information Management Institute of Healthcare Information Management National Chung Cheng University Chiayi County Taiwan; 3 Department of Neurology Tainan Sin Lau Hospital Tainan Taiwan; 4 School of Pharmacy Institute of Clinical Pharmacy and Pharmaceutical Sciences, College of Medicine National Cheng Kung University Tainan Taiwan; 5 Department of Information Management National Central University Taoyuan City Taiwan; 6 Center for Innovative Research on Aging Society National Chung Cheng University Chiayi County Taiwan; 7 MOST AI Biomedical Research Center National Cheng Kung University Tainan Taiwan

**Keywords:** administrative claims data, bibliometric analysis, National Health Insurance, text mining, open access journals, PubMed

## Abstract

**Background:**

Studies using Taiwan’s National Health Insurance (NHI) claims data have expanded rapidly both in quantity and quality during the first decade following the first study published in 2000. However, some of these studies were criticized for being merely data-dredging studies rather than hypothesis-driven. In addition, the use of claims data without the explicit authorization from individual patients has incurred litigation.

**Objective:**

This study aimed to investigate whether the research output during the second decade after the release of the NHI claims database continues growing, to explore how the emergence of open access mega journals (OAMJs) and lawsuit against the use of this database affect the research topics and publication volume and to discuss the underlying reasons.

**Methods:**

PubMed was used to locate publications based on NHI claims data between 1996 and 2017. Concept extraction using MetaMap was employed to mine research topics from article titles. Research trends were analyzed from various aspects, including publication amount, journals, research topics and types, and cooperation between authors.

**Results:**

A total of 4473 articles were identified. A rapid growth in publications was witnessed from 2000 to 2015, followed by a plateau. Diabetes, stroke, and dementia were the top 3 most popular research topics whereas statin therapy, metformin, and Chinese herbal medicine were the most investigated interventions. Approximately one-third of the articles were published in open access journals. Studies with two or more medical conditions, but without any intervention, were the most common study type. Studies of this type tended to be contributed by prolific authors and published in OAMJs.

**Conclusions:**

The growth in publication volume during the second decade after the release of the NHI claims database was different from that during the first decade. OAMJs appeared to provide fertile soil for the rapid growth of research based on NHI claims data, in particular for those studies with two or medical conditions in the article title. A halt in the growth of publication volume was observed after the use of NHI claims data for research purposes had been restricted in response to legal controversy. More efforts are needed to improve the impact of knowledge gained from NHI claims data on medical decisions and policy making.

## Introduction

Health care administrative data, also known as administrative claims data, [1], are derived from claims for reimbursement for routine health care services. They are relatively inexpensive to procure and, in general, readily available in electronic format [2]. Therefore, they are widely used for medical and public health research [1,3,4]. Such claims-based studies may cover a variety of research types, such as disease surveillance, health service utilization, validity analysis, and association between exposure and health outcomes [5].

Taiwan’s National Health Insurance Research Database (NHIRD), one of the largest health care administrative databases in the world, has provided a great opportunity for researchers to perform population-based studies [6]. Since 1995, residents in Taiwan have enjoyed a universal single-payer health care system operated by the National Health Insurance (NHI). The program covered virtually all of Taiwan’s population (99.5%) by 2010. The coverage of the whole population in the database has the advantages of an enormous sample size and lack of participation bias [6]. In 2000, Taiwan’s National Health Research Institutes compiled NHI claims data into the NHIRD and made it publicly available to the academic community. Low-cost updates of the NHIRD are possible because its data collection is carried out routinely for purposes inherent in the process of medical care and insurance reimbursement [6]. The public release of this large population-based database enables collaboration and knowledge sharing among researchers and boosts scientific production [6,7]. A bibliometric study conducted by Chen et al [8] to investigate the trend of using the NHIRD as research material found that the number of these studies had increased significantly from 2000 to 2009, with an average annual growth rate of 45.8%.

While more and more authors have successfully published studies using NHI claims data as the data source, some of these studies were criticized for being merely data-dredging studies rather than hypothesis-driven [9]. For example, as described by Hampson et al [9], increased risks of 5 different medical conditions following carbon monoxide poisoning were reported in 5 individual articles using the same research model. In addition, the emergence of open access mega journals (OAMJs) since 2006 [10] may have played a role. OAMJs, as a new business model for publication, are characterized by a large publication volume and broad disciplinary scope. They generally accept research articles as long as the requirements of “scientific soundness” are met, regardless of their contributions to a research field or academic interest [10]. It is believed, justified or not, that Journal Citation Reports (JCR)-indexed OAMJs are especially attractive to some researchers in Taiwan [10,11].

On the other hand, with the widespread use of the NHIRD in research, several human rights groups protested the use of claims data without the explicit authorization from individual patients and launched a lawsuit against the NHI Administration in 2012 [12,13]. As a result, the National Health Research Institutes stopped accepting applications for the NHIRD from researchers at the end of 2015 and terminated the NHIRD service in mid-2016 [14]. Thereafter, researchers can only access NHI claims data on site at the Data Science Center or via a virtual private network connection at its branch offices [15].

Studies based on the NHIRD have expanded substantially in both quantity and quality during the period of 2000 to 2009 [8]. However, whether the recently burgeoning OAMJs or lawsuit would affect the research output from NHI claims data remained undetermined. Since Asian countries like Japan and South Korea are also developing their own nationwide claim databases [16], the lessons regarding how Taiwan’s NHI claims database contributed to academic production may help researchers in other countries to develop their own policy and strategy on how to use claims databases in research. Accordingly, we formulated the following research questions: (1) With the widespread use of NHI claims data as research material, does the number of research articles in the second decade keep growing at a similar pace to that observed in the first decade since the release of NHI claims data? What is the effect of the lawsuit against the secondary use of health insurance data for research on research output? (2) What are the most common research topics of articles using NHI claims data? Do the types of research topics correlate with the volume of research output? (3) Which journals published the most articles using NHI claims data? (4) What is the role of open access journals in the proliferation of research output? (5) Who are the most prolific authors and research groups?

## Methods

### Data Source

This study used PubMed to locate publications that may have used NHI claims data as the primary data source because PubMed is the most widely used database for searching medical literature. Articles published in English that entered PubMed between Jan 1, 1996 and Dec 31, 2017 with “journal article” as their publication type were included. Following the work by Chen et al [8], a broad search strategy was employed to permit inclusion of as many articles as possible. Articles had to mention “Taiwan” in any of the textual fields including title, abstract, medical subject heading (MeSH) index terms, and author’s affiliation address and meet any of the following criteria: (1) indexed with the MeSH term “insurance, health” or “national health programs;” (2) either “nationwide” or “population” in the title field; (3) any of the following terms appearing in the title or abstract: “health insurance,” “national insurance,” “claims data*,” “claim data*,” “insurance claim*,” “insurance data*,” “administrative data*,” “nationwide data*,” “national data*,” “NHIRD,” “LHID,” “NHI,” and “BNHI.” The asterisk (*) is the truncation symbol used by PubMed that indicates to find all terms that begin with the string preceding the asterisk. Articles classified under the categories of comment, letter, editorial, or review and those published without an abstract were excluded. The search was done on June 24, 2018 and resulted in 5105 articles for further evaluation (See Multimedia Appendix 1).

Because journals may change their titles or even merge, this study always adopted the last title of a journal. JCR Science Edition and Social Sciences Edition (Clarivate Analytics, 2018) was used to retrieve 2017 Journal Impact Factors and journal categories. Journals were classified from Q1 to Q4 according to the impact factor quartiles in the specific journal category, where Q1 journals stand for journals with higher impact factors. Journals not indexed by the JCR were classified as other. Open access journals were identified through the Directory of Open Access Journals. OAMJs were defined as described in a previous study [10].

### Ascertainment of Studies

All the articles were downloaded from PubMed and preprocessed using the “easyPubMed” package in R. Because the list of potential articles was quite lengthy, several heuristic rules were applied to determine whether an article used NHI claims data as the primary data source. Basically, regular expression pattern matching was used to detect the mentioning of using NHI claims data in article abstracts and adjusted the matching patterns by trial and error. This study finally found two inclusion rules and one exclusion rule. The rules are shown in Textbox 1. These rules identified 3059 articles as using NHI claims data and achieved 100% precision (positive predictive value) by manually examining a random sample of 500 articles.

The remaining 2046 articles were reviewed by the first and second authors. Each author independently classified an article as “using NHI claims data,” “not using NHI claims data,” or “using data from undetermined source” by examining its abstract or full text, when necessary. This process achieved an agreement of 99.1% (kappa=0.978), and discrepancies (19 articles) were resolved by consensus. Among them, 632 articles were considered not using NHI claims data and thus excluded. In the end, a total of 4473 articles were included in this study (Multimedia Appendix 1).

**Heuristic rules used to determine whether an article used National Health Insurance claims data.**

**Inclusion rules**

1. (from|data|study|using|cohort|used|based|patients|identified|population|obtained|

claim|conducted|retrieved|collected|selected|analyz)[[:print:]]{1,20}(National Health Insurance|NHI|Longitudinal Health Insurance|insurance claims|Registry for Catastrophic Illness Patient)[[:print:]]{1,20}(claim|data|file)”)

2. (National Health Insurance|NHI|Longitudinal Health Insurance|insurance claims|Registry for Catastrophic Illness Patients)[[:print;]]{1,20}(claim|data|file)[[:print:]]{1,20}(from|

used|patients|identified)

**Exclusion rule**

1. (Korea)[[:print:]]{1,20}(National Health Insurance|NHI)

### Text Mining

This study used MetaMap as the tool to mine medical entities, such as symptoms, clinical findings, diseases, and medications, from article titles. MetaMap is a natural language processing tool developed by the National Library of Medicine. It analyzes input text through tokenization, sentence boundary determination, part-of-speech tagging, and parsing and generates variants of resulting phrases or words [17]. By evaluating measures of centrality, variation, coverage, and cohesiveness, MetaMap locates each matched medical entity in the Unified Medical Language System (UMLS) Metathesaurus, assigns it a semantic type, and returns a concept unique identifier and score between 0 and 1000, with a higher value representing a closer match [18]. This study used UMLS Metathesaurus version 2016AA.

Because an article title typically indicates what the article is about, this study attempted to mine knowledge from article titles. Although MeSH terms are also used in PubMed to describe what an article is about, we analyzed MetaMap-derived concepts instead of MeSH concepts for two reasons. First, we focused on the article title, but the MeSH concepts were determined by examining the whole article; second, the UMLS Metathesaurus contains far more medical concepts than the MeSH vocabulary. This study focused on two categories of medical entities: (1) medical conditions including diseases, symptoms and signs, and findings and (2) interventions, including medications, procedures, and surgery. Specifically, medical entities were categorized based on their UMLS semantic types (see Multimedia Appendix 2). Because MetaMap may generate multiple UMLS semantic types and concepts from the same phrase [18], this study relied on the order output by MetaMap and accepted only the first returned semantic type and concept. Furthermore, MetaMap might retrieve general terms that are not the medical entities the researchers were interested in, such as “adopt,” “75+ years,” and “ambulatory visit”. Therefore, this study collected these terms in a stop word list by manually reviewing an aggregate list of the concepts returned by MetaMap. The terms in the stop word list were then excluded from analysis.

Researchers may have different preferences for research topics when using administrative databases as the primary data source [5]. Descriptive studies may investigate only one medical condition whereas analytical studies generally focus on the association between an exposure (either a medical condition or an intervention) and an outcome (a medical condition) [19]. Some studies that used NHI claims data were considered to replicate the same research model of examining the association between two medical conditions [9]. Motivated by this criticism, this study classified articles into 4 study types based on medical entities mentioned in the title as follows: (1) with ≥1 intervention regardless of the number of medical conditions, (2) with ≥2 medical conditions but without any intervention, (3) with only 1 medical condition but without any intervention, and (4) others. Each article was assigned to only 1 of the 4 study types. For example, in the article title “Tamoxifen and the risk of Parkinson's disease in female patients with breast cancer in Asian people,” tamoxifen is an intervention whereas Parkinson’s disease and breast cancer are both medical conditions. This article is classified as the type with ≥1 intervention. An article with the title “Increased risk of stroke in patients with chronic kidney disease after recurrent hypoglycemia” mentions three medical conditions and is classified as the type with ≥2 medical conditions but without any intervention.

In order to offer background statistics, PubMed was queried to identify the number of articles published in English that entered PubMed between 2000 and 2017 with “journal article” as their publication type. The number of articles published between 2000 and 2017 in the top 20 journals that have published the most studies using NHI claims data were also obtained. Furthermore, the titles of the articles in PubMed and the top 20 journals were screened for presence of the top 10 medical conditions and top 10 interventions that were the most prevalent among studies using NHI claims data. The percentage of each medical condition or intervention among published articles was calculated.

### Statistical Analysis and Data Visualization

Categorical variables are reported as counts (percentages). Comparisons between groups used chi-square tests. Trends in continuous variables were assessed using the Cuzick test. Trends in categorical outcomes were evaluated using the Cochran-Armitage trend test for binomial proportions and the multinomial Cochran-Armitage Trend Test implemented in the R package “multiCA” [20] for multinomial proportions. Social network analysis was applied to explore the cooperation between authors and the relationships between medical entities. The “igraph” package in R was used to produce the network graph. Each node represents an author or a medical entity. The nodes are joined by weighted links, in which the width of a link indicates the frequency of relationships between two nodes. The size of a node is proportional to the weighted degree centrality of the node, which is computed by summing the weights of links to the node.

Statistical analyses and visualizations were performed using Stata 15.1 (StataCorp, College Station, Texas) and R version 3.5.0 (R Foundation for Statistical Computing, Vienna, Austria). Two-tailed *P* values were considered statistically significant at <.05.

## Results

### Trends in Research Output

**Figure 1:**
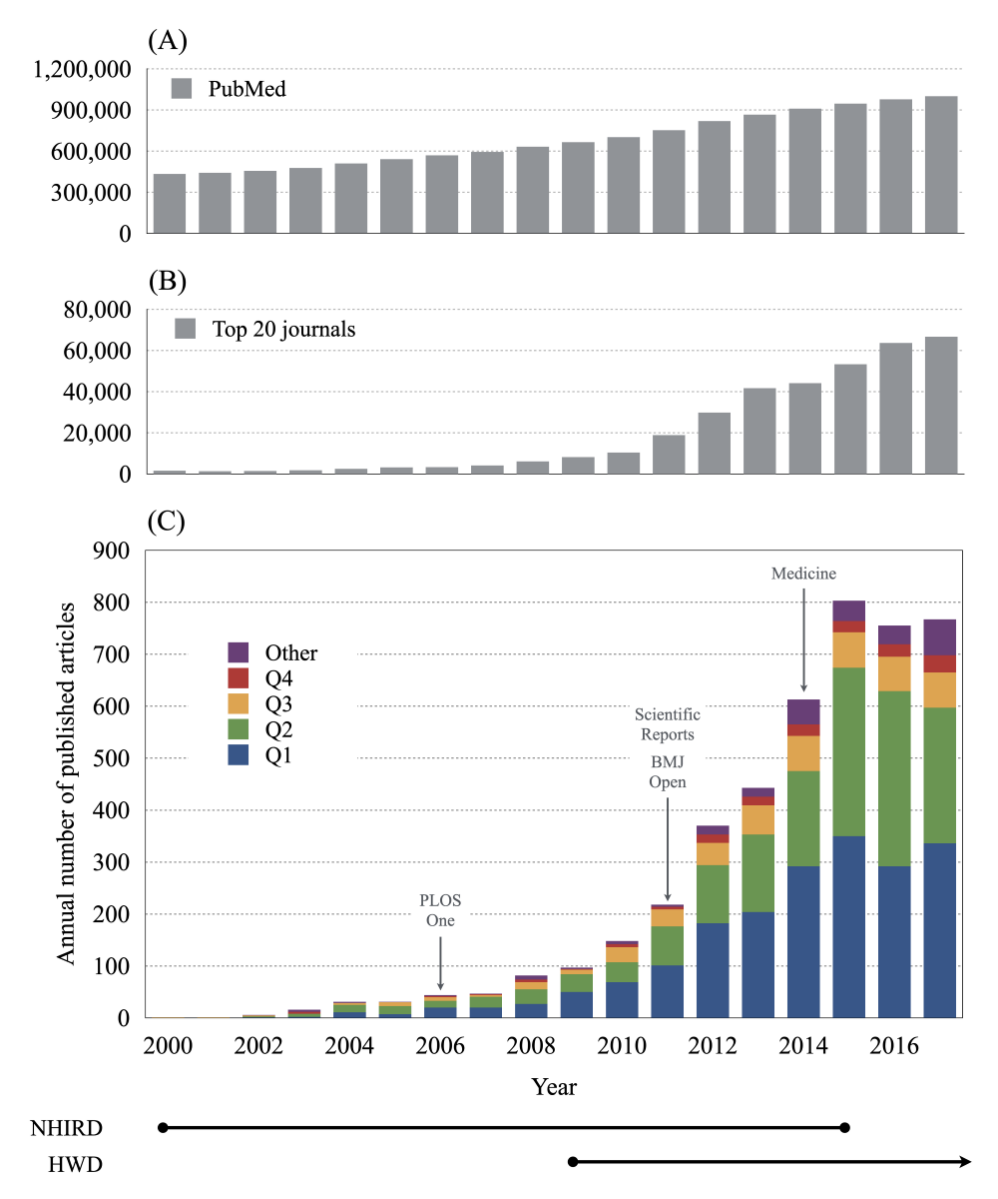
Annual number of publications between 2000 and 2017 (A) in PubMed, (B) in the top 20 journals that have published the most studies using National Health Insurance (NHI) claims data, and (C) based on NHI claims data, separated by JCR (2017 edition) ranking (Q1, Q2, Q3, Q4). The first year of each major OAMJ is indicated. The release of the National Health Insurance Research Databases (NHIRD) began in 2000 and ended in 2015. The Health and Welfare Database (HWD) was created in 2009 and is still available for research use.

Since the first article appeared in 2000, the number of publications grew tremendously until 2015, when the publication output seemed to reach a plateau (Figure 1). In contrast, the number of publications in PubMed and the top 20 journals that have published the most studies using NHI claims data continued to increase after 2015 (Figure 1). By 2015, the average annual growth rate of published articles was 77.4%, with a doubling time of 1.7 years. Almost all the articles (97.0%) were published in journals indexed in the JCR Science Edition or Social Sciences Edition. Among the articles indexed in the JCR, 46.4%, 38.3%, 11.5%, and 3.8% were published in Q1, Q2, Q3, and Q4 journals, respectively. Figure 1 illustrates the distribution of articles across the four quartiles of journals in the JCR each year. Table 1 gives the characteristics of articles, authors, and journals across 3 time periods and the results of the trend analysis by year. Based on the affiliation of the first author, we determined that researchers in nonhospital institutions published most of the articles initially, which were gradually outnumbered by those produced by hospital researchers in recent years. Articles were increasingly published in open access journals, in particular OAMJs. Articles were published in an increasing number of journals and were more widely distributed across disciplines.

**Table 1:** Characteristics of and trends for articles and journals.

Articles and journals		Period			Total number	*P* value for trend
		2000–2005	2006–2011	2012–2017		
**Articles**						
	Indexed in PubMed, n	86	636	3751	4473	<.001
	Indexed in JCR^a^ 2017, n (%)	78 (90.7)	625 (98.3)	3635 (96.9)	4338 (97.0)	.219
	First author from hospitals, n (%)	33 (38.4)	303 (47.6)	2479 (66.1)	2815 (62.9)	<.001
	First author from abroad	3 (3.5)	20 (3.1)	71 (1.9)	94 (2.1)	.019
	Published in OAJs^b^, n (%)	16 (18.6)	103 (16.2)	1447 (38.6)	1566 (35.0)	<.001
	Published in OAMJs^c^, n (%)	0 (0)	8 (1.3)	898 (23.9)	906 (20.3)	<.001
**Study types per article title**						<.001
	With ≥1 intervention, n (%)	29 (33.7)	202 (31.8)	1315 (35.1)	1546 (34.6)	.002
	With ≥2 conditions, n (%)	8 (9.3)	132 (20.8)	1435 (38.3)	1575 (35.2)	<.001
	With only 1 condition, n (%)	30 (34.9)	210 (33.0)	797 (21.2)	1037 (23.2)	<.001
	Others, n (%)	19 (22.1)	92 (14.5)	204 (5.4)	315 (7.0)	<.001
**Journals**						
	Indexed in PubMed, n	59	297	766	841	<.001
	Indexed in JCR 2017, n	53	287	727	791	<.001
	Journal categories, n	32	56	68	68	<.001

^a^JCR: Journal Citation Reports.

^b^OAJ: open access journal.

^c^OAMJ, open access mega journal.

### Research Topics of Articles

A total of 1763 medical entities were retrieved from article titles using MetaMap. Among these, 1132 entities belonged to the category of medical conditions, and 631 belonged to the category of interventions. The most commonly investigated medical conditions were diabetes (n=263), stroke (n=189), and dementia (n=139), whereas the most commonly studied interventions were statin (n=100), metformin (n=40), and Chinese herbal medicine (n=38). The top 50 medical conditions and interventions are listed in Table 2.

Table 3 gives the number and percentage of articles with one of the top 10 medical conditions or the top 10 interventions in the article title during the same period (2000 to 2017). Apparently, the top 10 medical conditions and top 10 interventions were not so prevalent in the titles among articles in PubMed or the top 20 journals.

**Figure 2:**
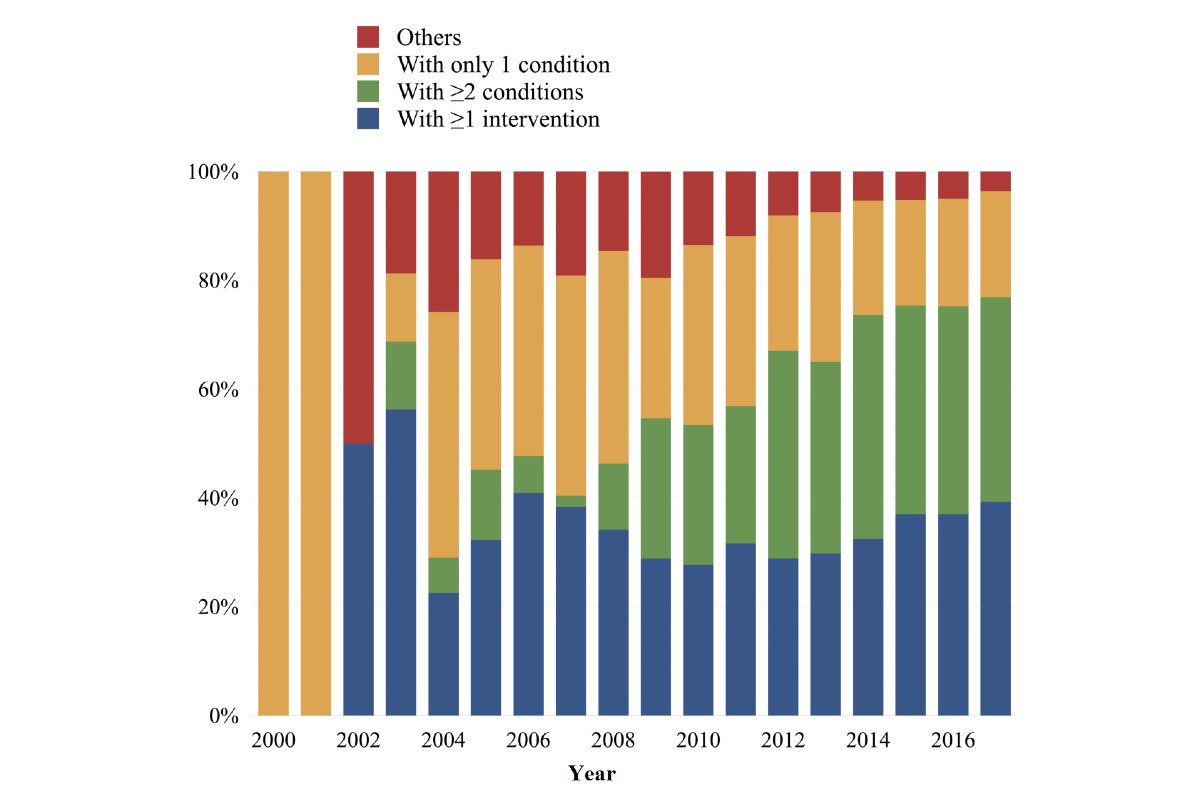
Distribution of study types across the years.

**Figure 3:**
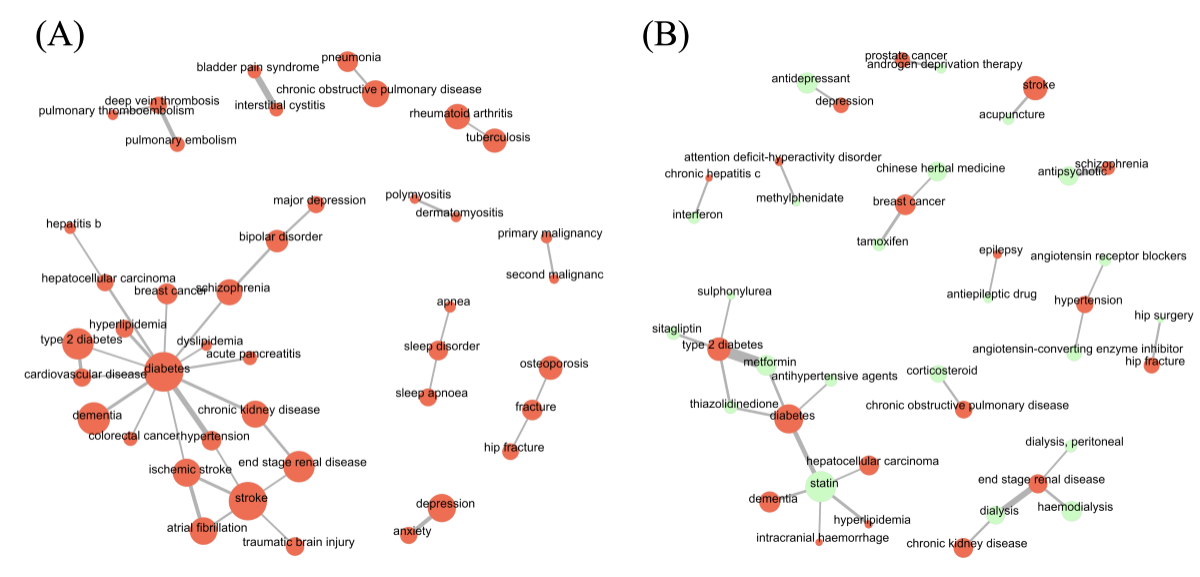
Network graphs displaying the most common (A) condition-condition pairs and (B) condition-intervention pairs.

Figure 2 illustrates the distribution of study types over the years. A significant trend in the proportions of study types was found (*P*<.001). In particular, the number of articles with ≥2 medical conditions in the title grew extensively whereas the number of articles with only one medical condition or without any medical entities in the title decreased considerably (Table 1). Figure 3 shows the most common condition-condition pairs and condition-intervention pairs in article titles. Diabetes, stroke, and dementia remained the top three medical conditions that were extensively studied in association with other medical conditions. They co-occurred with 118, 111, and 80 other medical conditions in article titles, respectively. Among the most studied interventions, statin, metformin, and Chinese herbal medicine co-occurred with 70, 31, and 28 medical conditions in article titles, respectively.

**Table 2:** Relative frequency of the top 50 medical conditions and interventions mentioned in article titles of studies based on National Health Insurance (NHI) claims data.

Medical conditions and interventions		Number of times mentioned
**Medical conditions**		
	Diabetes	263
	Stroke	189
	Dementia	139
	Type 2 diabetes	132
	Cancer	124
	End stage renal disease	102
	Atrial fibrillation	88
	Chronic obstructive pulmonary disease	88
	Chronic kidney disease	87
	Schizophrenia	82
	Ischemic stroke	80
	Asthma	79
	Depression	74
	Rheumatoid arthritis	71
	Breast cancer	68
	Tuberculosis	65
	Parkinson disease	59
	Acute myocardial infarction	55
	Bipolar disorder	55
	Hepatocellular carcinoma	55
	Hypertension	55
	Osteoporosis	55
	Hip fracture	54
	Lupus erythematosus, systemic	54
	Pneumonia	53
	Fracture	51
	Attention deficit-hyperactivity disorder	49
	Acute pancreatitis	46
	Cardiovascular disease	45
	Erectile dysfunction	41
	Lung cancer	41
	Acute coronary syndrome	40
	Coronary artery disease	38
	Depressive disorder	38
	Infection	37
	Peripheral arterial disease	37
	Prostate cancer	37
	Epilepsy	36
	Traumatic brain injury	36
	Sleep disorder	35
	Colorectal cancer	34
	Migraine	34
	Psoriasis	34
	Hearing loss, sudden	31
	Gout	27
	Liver abscess, pyogenic	27
	Obstructive sleep apnea	27
	Sleep apnea	27
	Alzheimer's disease	26
	Psychiatric disorder	26
**Interventions**		
	Statin	100
	Metformin	40
	Chinese herbal medicine	38
	Hemodialysis	38
	Antidepressant	36
	Antipsychotic	35
	Proton pump inhibitor	29
	Dialysis	27
	Nonsteroidal anti-inflammatory drugs	27
	Corticosteroid	24
	Influenza vaccination	24
	Angiotensin-converting enzyme inhibitor	22
	Benzodiazepine	21
	Zolpidem	19
	Antihypertensive agents	18
	Dialysis, peritoneal	17
	Thiazolidinedione	17
	Antidiabetic	16
	Angiotensin receptor blockers	14
	Reduction	14
	Tamoxifen	14
	Antibiotic	13
	Cholecystectomy	13
	Sitagliptin	13
	Angiotensin 2 receptor blockers	12
	Antiepileptic drug	12
	Chemotherapy	12
	Selective serotonin reuptake inhibitors	12
	Acupuncture	11
	Intervention, percutaneous coronary	11
	Mechanical ventilation	11
	Pioglitazone	11
	Appendectomy	10
	Aspirin	10
	Clopidogrel	10
	Hormone therapy	10
	Interferon	10
	Splenectomy	10
	Total knee arthroplasty	10
	Antiplatelet agents	9
	Caesarian section	9
	Coronary artery bypass grafting	9
	Drug eluting stent	9
	Liver transplantation	9
	Radiotherapy	9
	Resection	9
	Alendronate	8
	Antiviral	8
	Digoxin	8
	Hypnotic	8

**Table 3:** Number and percentage of articles with the corresponding condition or intervention in the article title between 2000 and 2007.

Articles		Studies using NHI^a^ claims data (n=4473), n (%)	Articles in top 20 journals n=362,463, n (%)	Articles in PubMed n=12,309,239, n (%)
**Articles with the condition in the article title**				
	Diabetes	263 (5.9)	4195 (1.2)	112,484 (0.9)
	Stroke	189 (4.2)	1944 (0.5)	56,781 (0.5)
	Dementia	139 (3.1)	664 (0.2)	24,217 (0.2)
	Type 2 diabetes	132 (3.0)	1724 (0.5)	38,757 (0.3)
	Cancer	124 (2.8)	22,030 (6.1)	503,283 (4.1)
	End stage renal disease	102 (2.3)	169 (0.0)	4387 (0.0)
	Atrial fibrillation	88 (2.0)	1189 (0.3)	21,867 (0.2)
	Chronic obstructive pulmonary disease	88 (2.0)	450 (0.1)	10,356 (0.1)
	Chronic kidney disease	87 (1.9)	671 (0.2)	12,617 (0.1)
	Schizophrenia	82 (1.8)	1215 (0.3)	33,536 (0.3)
**Articles with the intervention in the article title**				
	Statin	100 (2.2)	319 (0.1)	5130 (0.0)
	Metformin	40 (0.9)	372 (0.1)	6408 (0.1)
	Chinese herbal medicine	38 (0.8)	158 (0.0)	674 (0.0)
	Hemodialysis	38 (0.8)	58 (0.0)	3175 (0.0)
	Antidepressant	36 (0.8)	696 (0.2)	7327 (0.1)
	Antipsychotic	35 (0.8)	337 (0.1)	5540 (0.0)
	Proton pump inhibitor	29 (0.6)	38 (0.0)	1105 (0.0)
	Dialysis	27 (0.6)	416 (0.1)	16,301 (0.1)
	Nonsteroidal anti-inflammatory drugs	27 (0.6)	2 (0.0)	260 (0.0)
	Corticosteroid	24 (0.5)	109 (0.0)	4475 (0.0)

^a^NHI: National Health Insurance.

### Scattering of Articles in Journals

Until the end of 2017, 4473 articles were published in 841 journals, with an average of 5.3 articles per journal. Among these journals, 791 were indexed in the JCR Science Edition or Social Sciences Edition and were spread across 68 disciplines. The journals PLOS ONE and Medicine ranked first and second, respectively, in the number of publications and yielded 18.0% (804/4473) of the articles, whereas 333 journals published only one article each. This study applied Bradford's law and divided journals into three groups by the rank of journals, with each group of journals publishing approximately the same number of articles (Table 4). The top 20 journals represented one-third of all articles. In contrast, 113 and 708 journals contributed to the next two one-third proportions, respectively. The ratio of journal numbers among these three groups is 20:113:708 (1:5.7:35.4), which is quite close to 1:5.7:5.7^2^ (32.5).

**Table 4:** Scattering of articles in journals.

Group	Journals (n=841), n	Articles (n=4473), n	Cumulative, n (%)	Description
Top third	20	1504	1504 (33.6)	Publishing 26-419 articles
Middle third	113	1315	2819 (63.0)	Publishing 8-25 articles
Bottom third	708	1654	4473 (100.0)	Publishing 1-7 articles

### Role of Open Access Mega Journals

Open access journals published 35% of the studies, whereas OAMJs published around one-fifth of the studies (Table 1). Half of the top 20 journals are open access journals, with 4 journals considered to be OAMJs (Table 5). The four OAMJs (ie, PLOS ONE, Medicine, Scientific Reports, and BMJ Open) ranked first, second, seventh, and ninth, respectively, in terms of the number of publications. Table 6 shows the distribution of study types per article title across journal types. The proportions of study types were similar between open access journals and non-open access journals (*P*=.266). In contrast, OAMJs had a different distribution of study types than non-OAMJs (*P*<.001). They tended to publish articles with ≥2 medical conditions in the title.

**Table 5:** Top 20 journals ranked by published articles between 2000 and 2017.

Journal name	IF^a^	Rank in JCR^b^ in 2017	Articles, n (%)	OAJ^c^	OAMJ^d^
PLOS ONE	2.766	Multidisciplinary sciences (15/64)	419 (9.4)	Y	Y
Medicine	2.028	Medicine, general & internal (56/154)	385 (8.6)	Y	Y^e^
International Journal of Cardiology	4.034	Cardiac & cardiovascular systems (41/128)	72 (1.6)	N	N
Journal of the Formosan Medical Association	2.452	Medicine, general & internal (42/154)	52 (1.2)	Y	N
Oncotarget	N/A^f^	N/A	51 (1.1)	Y^g^	N
Journal of Affective Disorders	3.786	Clinical neurology (46/197), psychiatry (37/142), psychiatry^h^ (27/142)	51 (1.1)	N	N
Scientific Reports	4.122	Multidisciplinary sciences (12/64)	49 (1.1)	Y	Y
Pharmacoepidemiology and Drug Safety	2.314	Public, environmental & occupational health (66/180), pharmacology & pharmacy (145/261)	47 (1.1)	N	N
BMJ Open	2.413	Medicine, general & internal (43/154)	47 (1.1)	Y	Y
Journal of the Chinese Medical Association	1.660	Medicine, general & internal (72/154)	39 (0.9)	Y	N
Journal of Ethnopharmacology	3.115	Plant sciences (38/222); chemistry, medicinal (20/59); integrative & complementary medicine (4/27); pharmacology & pharmacy (87/261)	36 (0.8)	N	N
BMC Health Services Research	1.843	Health care sciences & services (53/94)	34 (0.8)	Y	N
European Journal of Internal Medicine	3.282	Medicine, general & internal (27/154)	30 (0.7)	N	N
Research in Developmental Disabilities	1.820	Education, special^h^ (8/40), rehabilitation^h^ (19/69)	30 (0.7)	N	N
Health Policy	2.293	Health care sciences & services (40/94), health policy & services^h^ (22/79)	29 (0.6)	N	N
Osteoporosis International	3.856	Endocrinology & metabolism (40/143)	28 (0.6)	N	N
Evidence-based Complementary and Alternative Medicine	2.064	Integrative & complementary medicine (10/27)	27 (0.6)	Y	N
QJM	3.204	Medicine, general & internal (30/154)	27 (0.6)	N	N
Journal of Clinical Psychiatry	4.247	Psychiatry (26/142), psychiatry^h^ (19/142), psychology, clinical^h^ (11/127)	26 (0.6)	N	N
International Journal of Environmental Research and Public Health	2.145	Environmental sciences (116/241); public, environmental & occupational health (73/180); public, environmental & occupational health^h^ (44/156)	25 (0.6)	Y	N

^a^IF: impact factor.

^b^JCR: Journal Citation Reports.

^c^OAJ: open access journal.

^d^OAMJ: open access mega journal.

^e^Converted to an OAMJ in 2014.

^f^N/A: not available.

^g^Not listed in the Directory of Open Access Journals.

^h^Social Sciences Edition.

**Table 6:** Distribution of study types per article title across journal types.

Study type	Open access journal, n (%)		Open access mega journal, n (%)	
	Yes (n=1566)	No (n=2907)	Yes (n=906)	No (n=276)
With ≥1 intervention	552 (35.2)	994 (34.2)	308 (34.0)	1238 (34.7)
With ≥2 conditions	537 (34.3)	1038 (35.7)	400 (44.2)	1175 (32.9)
With only 1 condition	353 (22.5)	684 (23.5)	159 (17.5)	878 (24.6)
Others	124 (7.9)	191 (6.6)	39 (4.3)	276 (7.7)

### Prolific Authors and Research Groups

**Figure 4:**
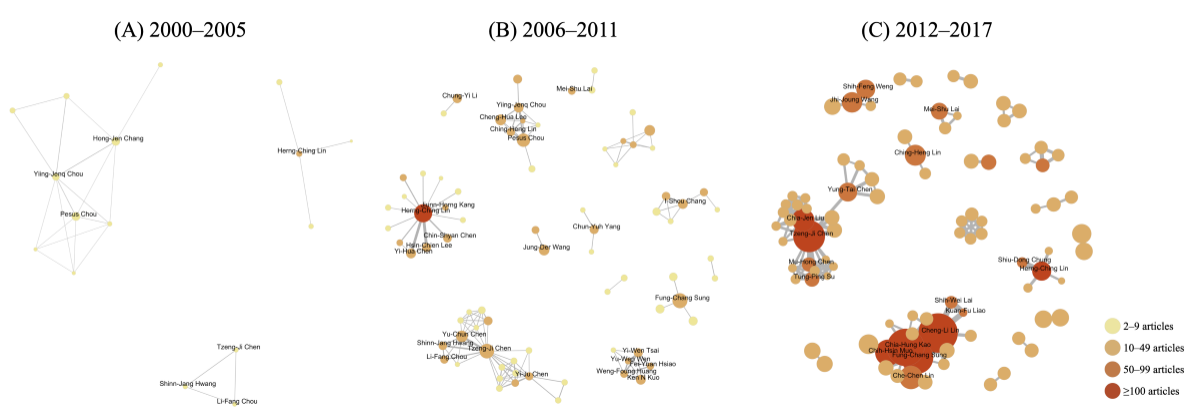
Co-authorship networks during (A) 2000-2005, (B) 2006-2011, and (C) 2012-2017.

The visualization in Figure 4 presents the networks of co-authorships during 2000 to 2005, 2006 to 2011, and 2012 to 2017. To avoid cluttering the figure, only author pairs who had collaborations in more than 2, 5, and 20 articles in the 3 time periods, respectively, were depicted. From 2000 to 2005, the social network analysis identified three main research groups. Herng-Ching Lin from Taipei Medical University was the most productive author and published at least 10 articles. Between 2006 and 2011, Herng-Ching Lin still produced the highest volume of publications (≥100). However, several other research groups emerged during this period. From 2012 to 2017, a total of 7 productive authors were identified, and each published at least 100 articles. Two large research groups can be readily observed in the network graph, including authors mainly from China Medical University Hospital (Cheng-Li Lin, Chia-Hung Kao, Fung-Chang Sung, et al) and Taipei Veterans General Hospital (Tseng-Ji Chen, Chia-Jen Liu, et al).

When a prolific author was defined as one who had at least 100 articles published between 2000 and 2017, a total of 8 authors were qualified as prolific. Table 7 shows that study types were significantly different between studies authored by at least one of the prolific authors and those that were not (*P*<.001). The most common type of studies contributed by prolific authors were studies with two or more medical conditions in the article title, whereas studies by nonprolific authors were more likely to mention at least one intervention in the article title.

**Table 7:** Distribution of study types per article title between studies authored by prolific authors and those authored by others.

Study type	Prolific author (≥100 articles), n (%)	
	Yes (n=1399)	No (n=3074)
With ≥1 intervention	385 (27.5)	1161 (37.8)
With ≥2 conditions	707 (50.5)	868 (28.2)
With only 1 condition	250 (17.9)	787 (25.6)
Others	57 (4.1)	258 (8.4)

## Discussion

### Principal Findings

This study yielded several interesting findings. First, a rapid growth in publications was observed from 2009 to 2015, just as it was between 2000 and 2009. However, the growth dramatically ceased after 2015. Second, certain medical conditions, such as diabetes, stroke, and dementia, and certain interventions such as statin therapy, metformin, and Chinese herbal medicine, received more attention from researchers using NHI claims data as the study material. Third, almost all the studies were published in JCR-indexed journals, most ranking as Q1 or Q2 in their corresponding JCR categories. OAMJs appeared to provide fertile soil for the rapid growth of research based on NHI claims data, particularly studies with ≥2 medical conditions in the article title. Fourth, while the top 8 most prolific authors contributed nearly one-third of all studies, they published more studies with ≥2 medical conditions in the article title than nonprolific authors. These studies mainly investigated the association between two medical conditions and might be easier to conduct than studies examining the effect of an intervention on a medical condition.

### Publication Volume

As described by Chen et al [8], the growth of literature using NHI claims data generally followed the proposed model of scientific growth [21] before 2015. The sudden halt of the growth trend after 2015 is quite unexpected. Although the underlying reasons are not fully understood, it is speculated to be related to the 2012 lawsuit against the secondary use of health insurance data [12,13]. Later, in reaction to the legal controversy, more precautionary measures for accessing NHI claims data were enforced [13]. Consequently, the National Health Research Institutes stopped the acceptance of new applications for procuring data from the NHIRD from December 2015 onwards. Moreover, permission to use a local copy of the NHIRD typically expires after 3 years. Therefore, after December 2018, researchers were allowed to access NHI claims data, which is now a part of the Health and Welfare Database, only within the Data Science Center of the Ministry of Health and Welfare or via a virtual private network from local branch offices of the Data Science Center across the country [22]. These measures definitely increased the barriers to conducting research using the NHI claims data. The question of whether the research output will decline from the present level awaits further observation.

### Research Topics

Diabetes, stroke, and dementia represented the most commonly investigated medical conditions. All these conditions are highly prevalent diseases that naturally attract more attention from researchers. Furthermore, their high prevalence enabled researchers to study these diseases using merely the Longitudinal Health Insurance Database, which is the 1 million–person subset of the NHIRD that entails a lower cost than the whole dataset of the NHIRD. In particular, because the diagnostic codes for diabetes and stroke have been validated within NHI claims data [23-25], researchers might be more confident performing research on these diseases. This again emphasizes the importance of case validation in secondary data analysis [26].

As for interventions, statins and metformin are commonly prescribed to patients with stroke and diabetes, respectively. Naturally, they were among the most frequently investigated interventions. In addition, the pleiotropic effects of statins and metformin might also intrigue researchers to test their effects on other diseases using large health care databases like the NHIRD [27,28]. Last but not least, the NHI program reimburses Chinese herbal medicine; therefore, NHI claims data provide researchers a unique opportunity to study the effectiveness of Chinese herbal medicine [29].

### Implications

Writing for publication is essential for academics. Currently, not only are academics evaluated against how well they publish but universities are also ranked according to their academic publication rates. The long-existing “publish or perish” culture of academia has now prevailed in Taiwan’s hospitals. Taiwan’s hospital accreditation system, in addition to assessing the quality of health care, also aims to determine the teaching status of a hospital [30]. Therefore, hospitals are putting more pressure on their staff to publish in JCR-indexed journals in order to meet the accreditation standards. The pressure to publish is reflected in the increasing trend of first authors coming from hospitals (Table 1).

In addition to these internal factors, the external environment is just suitable for catalyzing the growth of publications. The increasing availability of open access journals, in particular OAMJs, provides unprecedented capacity to accommodate a large volume of publications. Furthermore, several OAMJs (eg, PLOS ONE, Medicine, Scientific Reports, and BMJ Open) have decent impact factors and above-average JCR ranking (Q1 or Q2). All these factors have driven researchers to utilize secondary data analysis to augment their research output. Although the current system might have misdirected some hospital practitioners to “shallow research,” it has also encouraged positive involvement of practitioners in academic research.

Based on the text mining analysis, prolific authors tended to produce articles with ≥2 medical conditions in the title, while such articles were more likely to be published in OAMJs. From the pragmatic point of view, it is easier to investigate the association between two medical conditions than to study the effect of an intervention on a medical condition, in particular when the intervention, such as a medication, is time-dependent. Testing multiple hypotheses at the same time definitely increases the likelihood of finding an association [31]. Therefore, conducting studies investigating the association between two medical conditions within a large database appears to be a shortcut to increase research output. A typical approach is to examine whether condition A increases the risk of condition B. Some of these studies are criticized as “templated and non-hypothesis driven” and have raised concerns and disputes regarding the misuse of data analysis [9,32,33]. In addition, due to Berkson’s bias, such association studies may generate significant but spurious associations caused by inappropriate conditional factors such as hospitalization [34]. All these criticisms may give an impression that findings from studies using NHI claims data are useless to either clinical practitioners or policy makers.

### Future Directions

Despite the negative impression, the following strategies were proposed to increase the impact of research based on NHI claims data. First, the percentage of ﬁrst authors who are not Taiwanese citizens was very low (Table 1). As compared to studies using the United Kingdom’s electronic health database [35], international collaboration is relatively uncommon in studies using NHI claims data. In addition to seeking collaboration with foreign partners, the integration of databases from multiple countries can provide opportunities to compare disease prevalence and treatment effects across countries [36], hopefully producing more generalizable knowledge. Second, apart from formulating a hypothesis before conducting a study, researchers should focus on generating research questions with a real impact on clinical decision making rather than producing articles acceptable by journals with an impact factor. In this respect, studies using the United Kingdom’s electronic health database have set good examples of providing real-world evidence to inform clinical practice [37]. Future research should be directed towards clinically relevant and actionable study outcomes. Third, NHI claims data, like other administrative claims data, have been questioned about their data validity because claims data typically lack clinical information. Record linkage between NHI claims data and various clinical registries may be a promising approach to complement the advantages of each data source. The combination of detailed clinical information from registry databases and long-term outcome data from claims databases offers opportunities to enhance the validity of outcomes research [38]. Finally, as for the litigation from human rights groups against the use of NHI claims data, researchers should take this as an opportunity to meet the need for more dialogue and proactive participation from such groups.

### Limitations

This study has the following limitations. First, this study included only articles written in English and indexed in the PubMed database to make the results comparable with the study by Chen et al [8]. This undoubtedly limited the scope of this work to health-related publications. However, because the purpose of NHI claims data is to track payments for health care utilization, it is believed that most of these articles, if not all, were published in PubMed-indexed journals. Second, even though journal rankings change year by year, this study used 2017 JCR Journal Impact Factors and journal categories for journal ranking to simplify the analysis. Third, the analysis of article titles was based on automated natural language processing algorithms provided by MetaMap. Although MetaMap can effectively extract medical concepts from biomedical texts, the semantic relationships between the identified medical concepts are not readily discernible [39]. For example, it was difficult to determine whether a study truly investigated the association between two medical conditions simply based on the co-occurrence of the two conditions in the article title.

### Conclusions

As Taiwan has recently become an aged society and is expected to become a super-aged society by 2025 [40,41], health care expenditure will inevitably increase as the size of the elderly population grows. Therefore, knowledge of the burden of various diseases as well as the cost effectiveness of different diagnostic and treatment strategies is of paramount importance. Although nationwide disease registration and population-based surveys can provide valuable information to facilitate medical decisions and policy making, the processes of registration and surveys may themselves entail extra costs. In this regard, analysis of secondary data, such as NHI claims data, may continue to provide an affordable, alternative means of gaining knowledge.

## Appendix

Multimedia Appendix 1 Flowchart of included articles.

Multimedia Appendix 2 Unified Medical Language System (UMLS) semantic types for categorizing medical entities.

## Abbreviations

JCR: Journal Citation Reports.
MeSH: medical subject heading.
NHI: National Health Insurance.
NHIRD: National Health Insurance Research Database.
OAJ: open access journal.
OAMJ: open access mega journal.
UMLS: Unified Medical Language System.
