# Supplementary figures and images for: Two Decades of Research Using Taiwan’s National Health Insurance Claims Data: Bibliometric and Text Mining Analysis on PubMed
Source: J Med Internet Res. 2020 Jun 16;22(6):e18457. doi: 10.2196/18457 (PMC7327589; doi:10.2196/18457)

**Figure.** Flowchart of included articles.

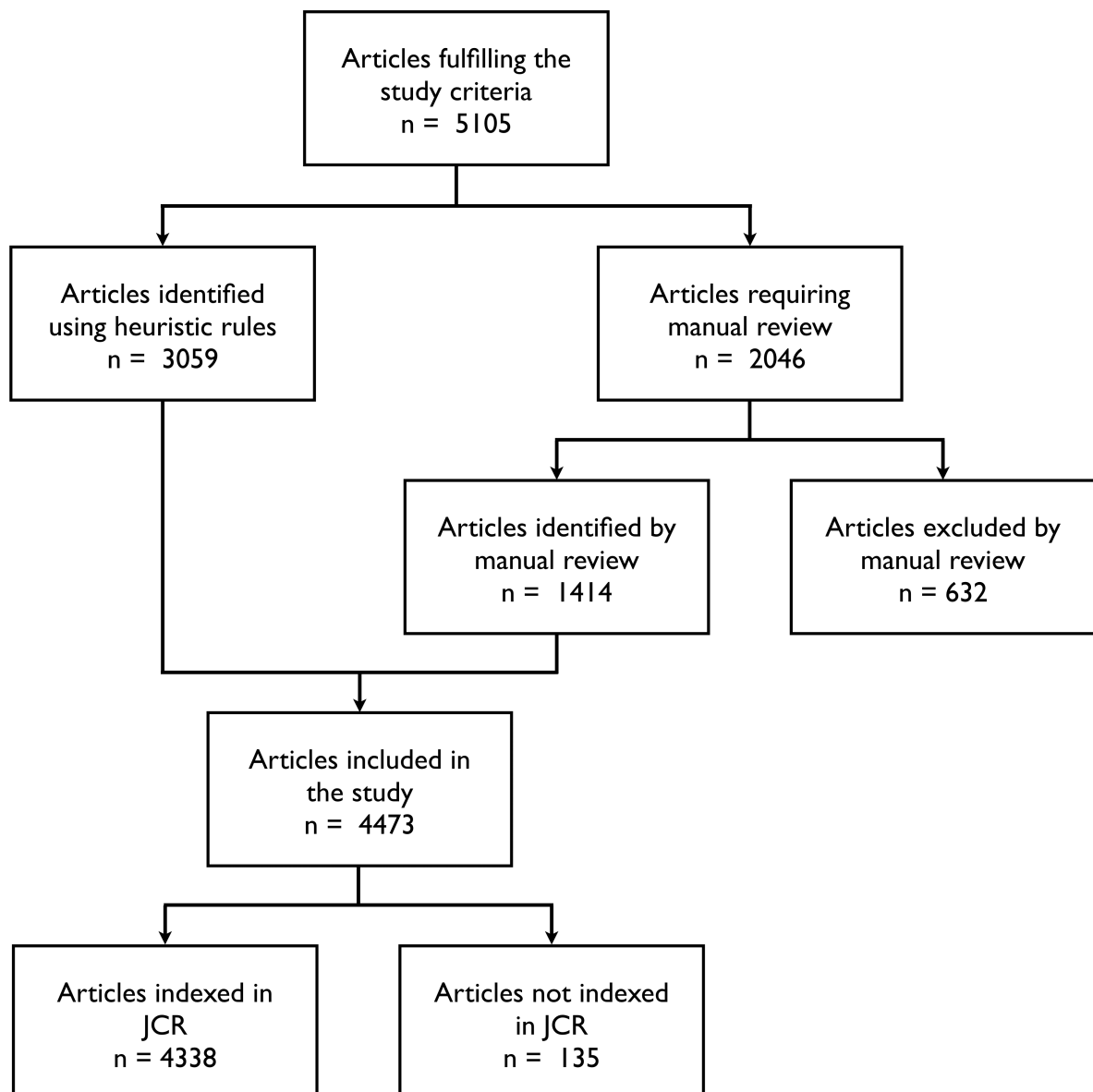

Supplement: Multimedia Appendix 1 [file jmir_v22i6e18457_app1.pdf]
