# Supplementary material for: Two Decades of Research Using Taiwan’s National Health Insurance Claims Data: Bibliometric and Text Mining Analysis on PubMed
Source: J Med Internet Res. 2020 Jun 16;22(6):e18457. doi: 10.2196/18457 (PMC7327589; doi:10.2196/18457)
Supplement: Multimedia Appendix 2 [file jmir_v22i6e18457_app2.pdf]

Table. UMLS semantic types for categorizing medical entities

Category	Semantic type	Abbreviation
Medical conditions	Acquired Abnormality	acab
	Anatomical Abnormality	anab
	Disease or Syndrome	dsyn
	Finding	fndg
	Injury or Poisoning	inpo
	Mental or Behavioral Dysfunction	mobd
	Neoplastic Process	neop
	Pathologic Function	patf
	Sign or Symptom	sosy
Interventions	Amino Acid, Peptide, or Protein	aapp
	Antibiotic	antb
	Biologically Active Substance	bacs
	Chemical	chem
	Clinical Drug	clnd
	Enzyme	enzy
	Hazardous or Poisonous Substance	hops
	Hormone	horm
	Immunologic Factor	imft
	Inorganic Chemical	inch
	Laboratory Procedure	lbpr
	Medical Device	medd
	Nucleic Acid, Nucleoside, or Nucleotide	nnon
	Organic Chemical	orch
	Pharmacologic Substance	phsu
	Therapeutic or Preventive Procedure	topp
	Vitamin	vita

UMLS, Unified Medical Language System.
